# Supplementary material for: CcNAC1 by Transcriptome Analysis Is Involved in Sudan Grass Secondary Cell Wall Formation as a Positive Regulator
Source: Int J Mol Sci. 2023 Mar 24;24(7):6149. doi: 10.3390/ijms24076149 (PMC10094045; doi:10.3390/ijms24076149)
Supplement: Supplementary file 1 [file ijms-24-06149-s001.zip › paper 6 Supplementary Figure.pdf]

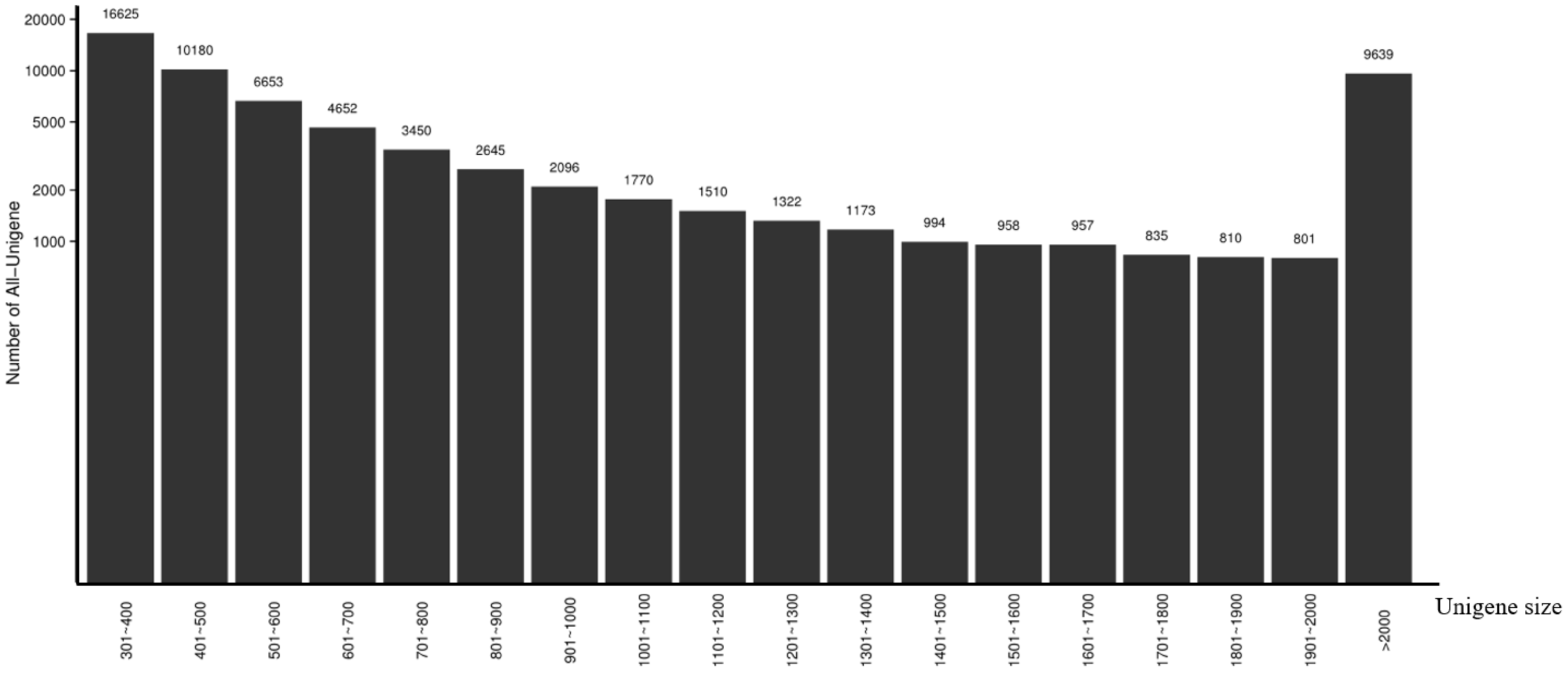

Supplementary Figure S1

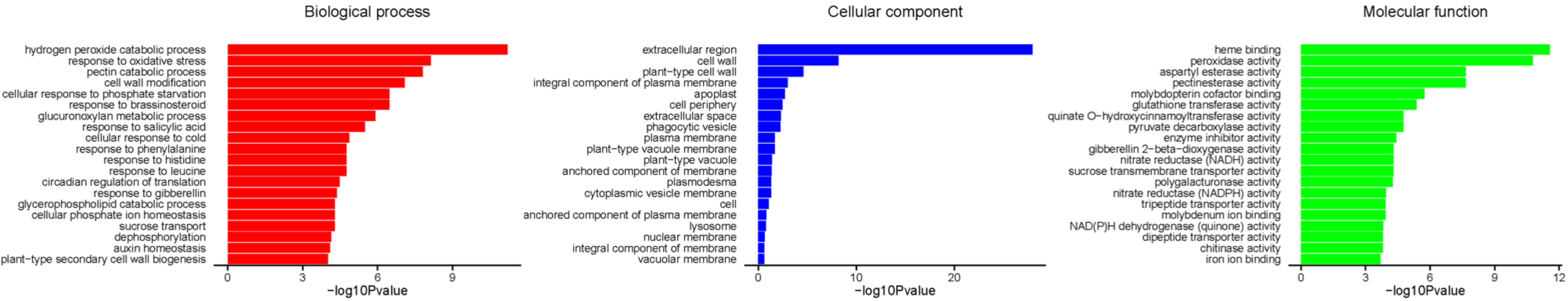

Supplementary Figure S2

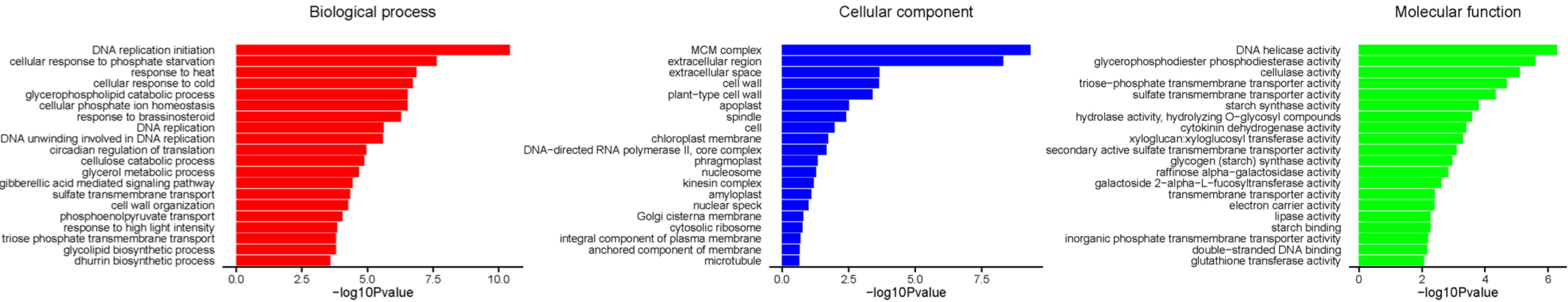

Supplementary Figure S3

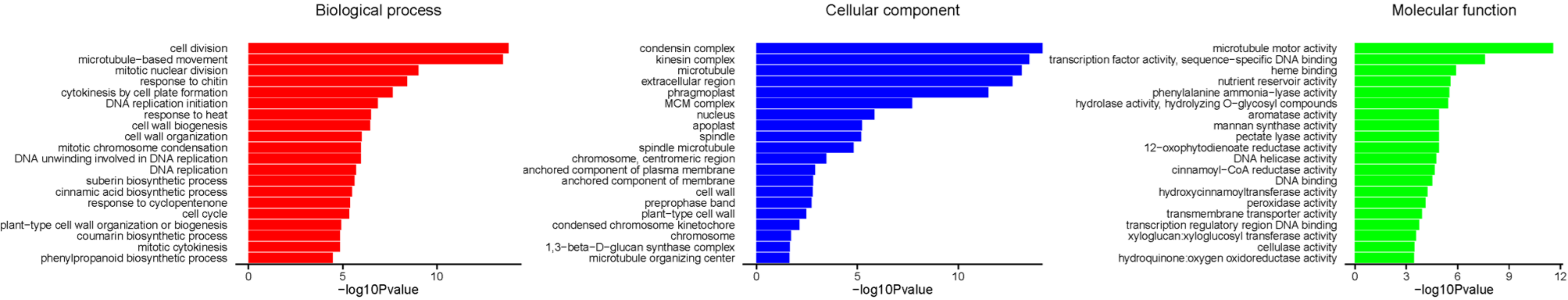

Supplementary Figure S4

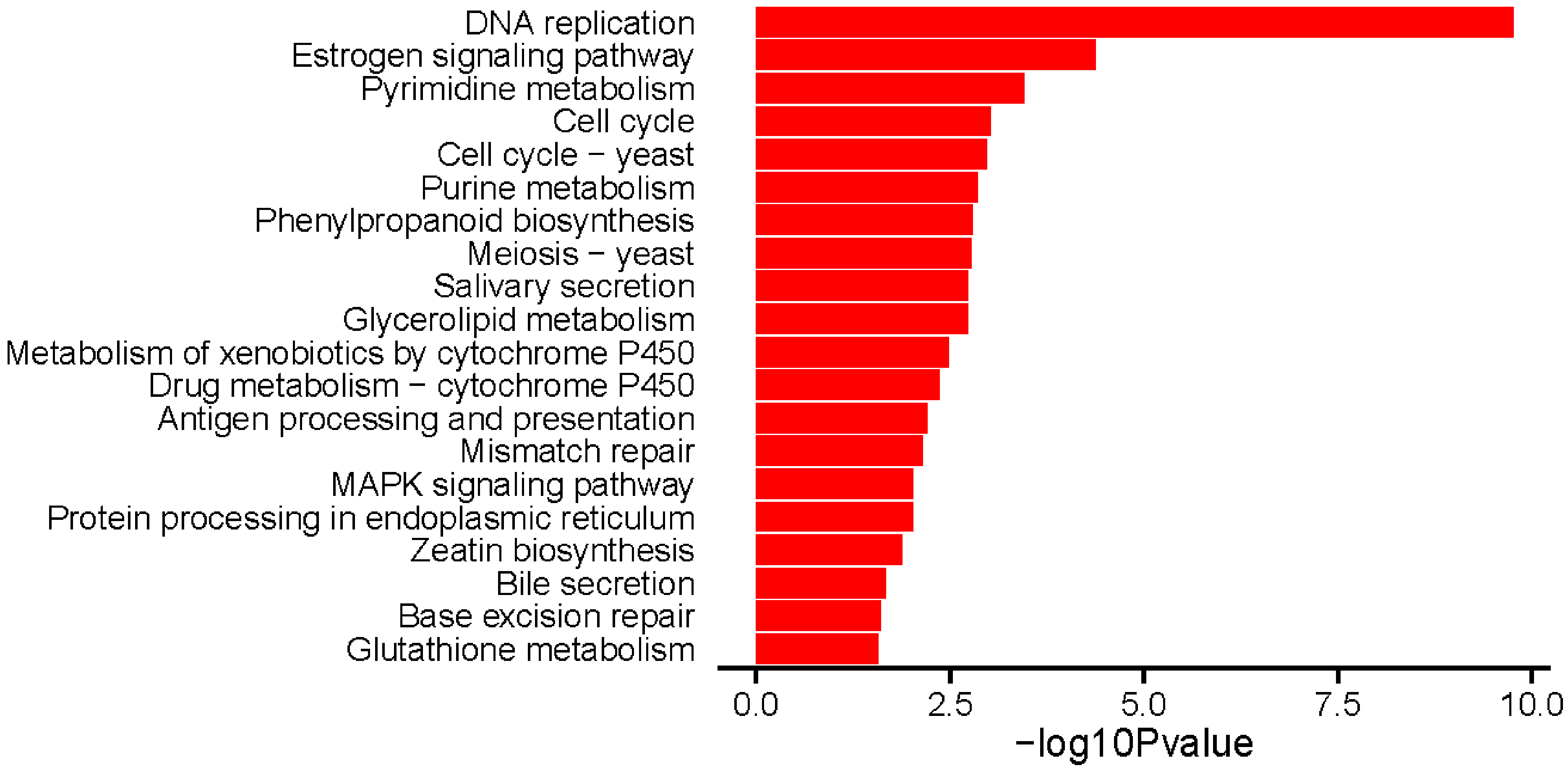

Supplementary Figure S5

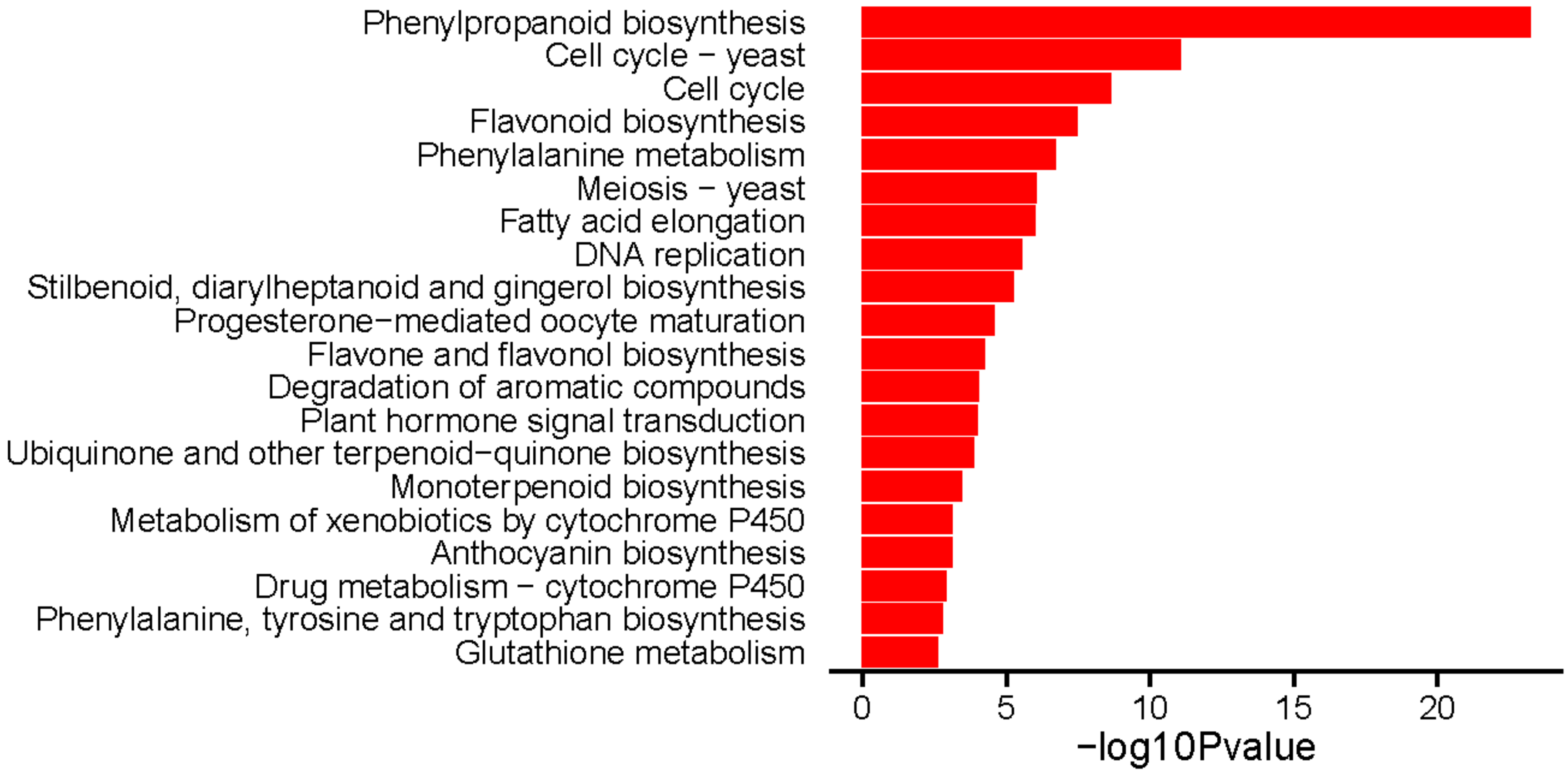

Supplementary Figure S6

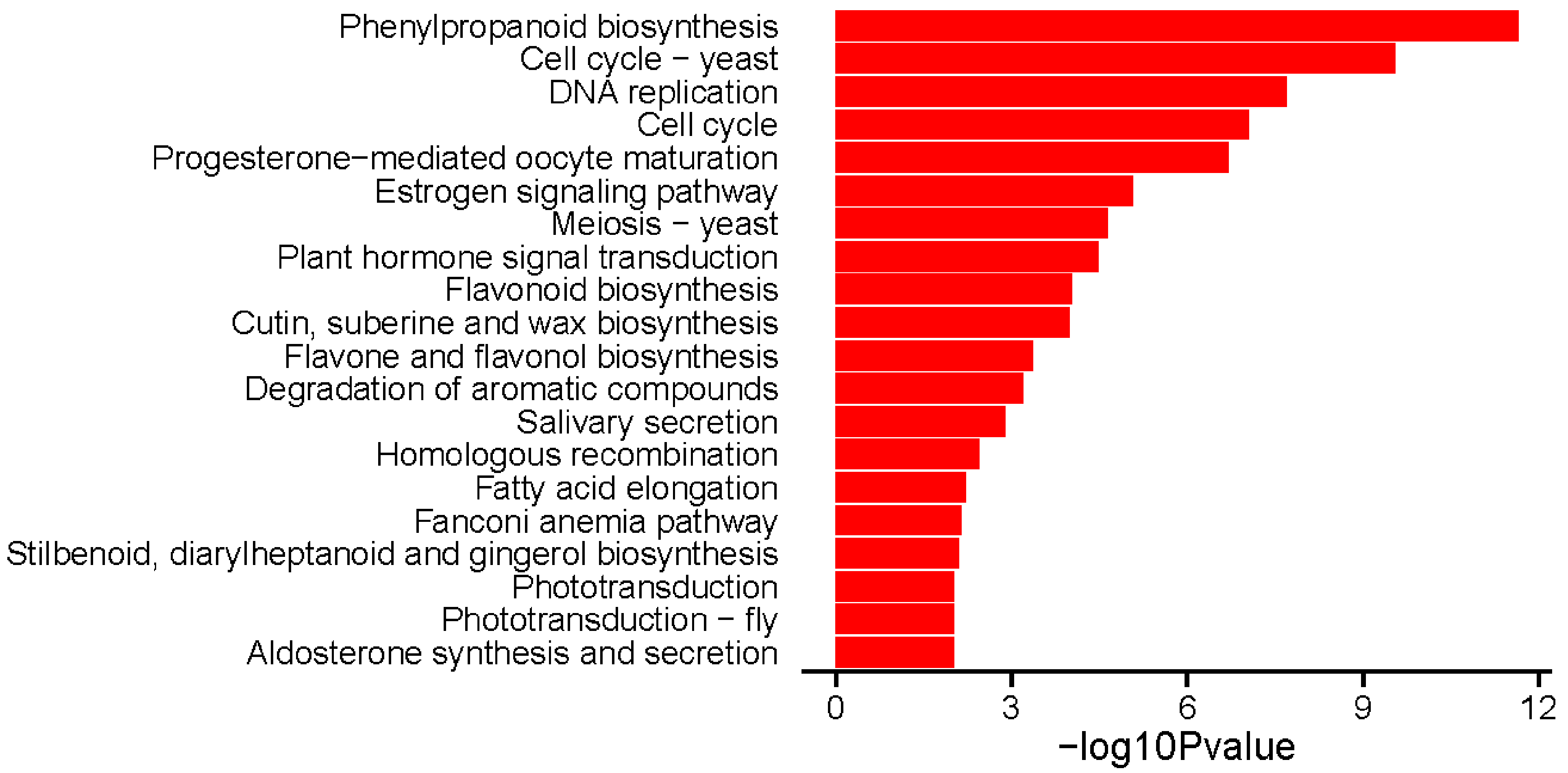

Supplementary Figure S7
