# Supplementary material for: CcNAC1 by Transcriptome Analysis Is Involved in Sudan Grass Secondary Cell Wall Formation as a Positive Regulator
Source: Int J Mol Sci. 2023 Mar 24;24(7):6149. doi: 10.3390/ijms24076149 (PMC10094045; doi:10.3390/ijms24076149)
Supplement: Supplementary file 1 [file ijms-24-06149-s001.zip › paper 6 Supplementary Tables S1&S2.pdf]

Supplemental Table S1 Transcriptome sequencing data result statistics

Sample	Raw reads	Raw bases	Clean reads	Clean bases	Valid ratio(base)	Q30(%)	GC content(%)
TEM+Dark18day	44383120	5547890000	44383120	5547408771	99.99%	95.66%	50.50%
TEMB2C5+Dark18day	44334420	5541802500	44334420	5541325729	99.99%	95.88%	51.00%
TEM+Light18day	44393360	5549170000	44393360	5548689544	99.99%	95.74%	52.50%
TEMB2C5+Light18day	44523400	5565425000	44523400	5564942324	99.99%	95.75%	51.00%
CK	44302640	5537830000	44302640	5537355253	99.99%	95.15%	50.50%

Supplemental Table S2

Primers used for clone gene

Gene	Sequence of primer pairs (5'-3')
CcNAC1	CCCTTCTCTCCTTCCTCCTCTTT/TTGCTGCTCTTCTACCCATGATG

Primers used for quantitative real-time PCR

Gene	Sequence of primer pairs (5'-3')
CcNAC1	GCGTGACAGGAAATACCCGA/CTGTACTCGTGCATGACCCA
CcEIF4a	AGGATTGGCACCAGAAGGGT/CACATCAAGCCCCTTGCAGA

Primers used to construct vector for plant expression and transgenic plants detection

Gene	Sequence of primer pairs (5'-3')
CcNAC1	GAGAACACGGGGGACTCTAGAATGGAGGAGGGGCTGCCG/
	AATGTTTGAACGATCGAGCTCTCAGAATGTTCTCCATGTCGACTC

Primers used to construct vector for subcellular localization

Gene	Sequence of primer pairs (5'-3')
CcNAC1	ATTTACGAACGATAGGGTACCATGGAGGAGGGGCTGCCG/
	TCCGTCGACCCCGGGGGTACCGAATGTTCTCCATGTCGACTCGT

Primers used to construct vector for yeast two-hybrid

Gene	Sequence of primer pairs (5'-3')
CcNAC1	TGGCCATGGAGGCCGAATTCATGGAGGAGGGGCTGCCGCCGG/
	CGCTGCAGGTCGACGGATCCTCAGAATGTTCTCCATGTCGACTCGTC
